# Editorial Comment to A case of laparoscopically assisted vulvar reconstruction using the gluteal fold flap for anterior enterocele after robot‐assisted radical cystectomy in a woman

**DOI:** 10.1002/iju5.12832

**Published:** 2025-01-22

**Authors:** Toru Kanno, Naoki Hayata

**Affiliations:** ^1^ Department of Urology National Hospital Organization Kyoto Medical Center Kyoto Japan

Vaginal complications following female radical cystectomy were once considered rare, but their increasing frequency in recent years has drawn significant attention. Reported complications include vaginal prolapse, vaginal fistula, dyspareunia, anterior enterocele, and vaginal cuff dehiscence. We previously reported vaginal cuff dehiscence in seven of 100 female patients (7%) who underwent laparoscopic radical cystectomy,^1^ highlighting the potential severity and life‐threatening nature of these complications. Interestingly, reports of vaginal complications have increased since minimally invasive surgery, including laparoscopic and robot‐assisted surgeries, became the standard approach, although the association remains unclear. This paper, which describes a flap‐based repair for anterior enterocele,^2^ is highly valuable.

The management of vaginal complications after radical cystectomy requires proficiency in both prevention and treatment strategies. The posterior vaginal wall is thinner than the anterior wall, suggesting that extensive resection of the anterior wall may increase the risk of complications. Therefore, preservation of the anterior vaginal wall should be prioritized, and organ‐sparing female radical cystectomy should be considered whenever oncologically permissible. Interestingly, retrograde dissection techniques to preserve the anterior vaginal wall have also been reported.^3^ It remains unclear whether other surgical or patient factors contribute to vaginal complications, and no clearly effective additional preventive measures are currently known.

When vaginal complications do occur, the choice of surgical repair becomes crucial. Surgical approaches can be broadly categorized into mesh‐based repairs^4^ and flap‐based repairs, such as the gluteal fold flap described in this case. Other flap options, including the gracilis myocutaneous flap, have also been reported.^5^ However, no consensus has been reached regarding the optimal repair technique. Future accumulation of cases will be essential to establish tailored repair methods for individual scenarios.

Above all, urologic surgeons performing radical cystectomy must remain highly aware of the potential for these complications and their impact on patients' postoperative outcomes.

## Conflict of interest

The authors declare no conflict of interest.
